# Toward More Predictable Musculoskeletal Embolization: A Translational Evaluation of Imipenem–Cilastatin Performance and Safety in a Porcine Model of Tendon Neovascularization

**DOI:** 10.3390/jpm16100487

**Published:** 2026-09-22

**Authors:** Pierre Julien Felix, Noelia M. Sanchez-Ballester, Marylène Bacle, Alix Pantel, Ian Soulairol, Julien Frandon

**Affiliations:** 1 IMAGINE UR UM 103, Department of Medical Imaging, Nîmes University Hospital, Montpellier University, 30029 Nîmes, France; 2 ICGM, Montpellier University, CNRS, ENSCM, 34293 Montpellier, Franceian.soulairol@chu-nimes.fr (I.S.); 3 Department of Pharmacy, Nîmes University Hospital, 30029 Nîmes, France; 4 RAM-PTNIM, Medical School of Nîmes, Montpellier University, 34090 Montpellier, France; marylene.bacle@umontpellier.fr; 5 INSERM U1047 VBIC, Department of Microbiology and Hospital Hygiene, Nîmes University Hospital, Montpellier University, 30029 Nîmes, France; alix.pantel@chu-nimes.fr

**Keywords:** musculoskeletal, personalized medicine, patient selection, precision embolization, translational interventional radiology

## Abstract

**Background:** There is a need to provide a translational evaluation of imipenem–cilastatin as an innovative musculoskeletal embolic agent by assessing its embolization efficacy, physicochemical behavior, and safety profile in a porcine model of intratendinous neovascularization. **Materials and Methods:** In vitro analyses were performed to assess size distribution and solubility of imipenem–cilastatin in iodinated contrast media (Visipaque^®^) for two commercial formulations (Tienam^®^ and Sun^®^). A preclinical study was conducted on six pigs, with neovascularization induced via collagenase injection in the patellar tendon. Each animal received Tienam^®^ embolization with Visipaque^®^ on one side and Visipaque^®^ alone on the contralateral side at D7 post induction, followed by angiographic evaluation at D14. Efficacy was assessed using a four-point neovascularization score. Safety was evaluated through behavioral monitoring and rectal flora analysis. **Results:** In vitro, imipenem–cilastatin exhibited heterogeneous particle morphology and size: D50 was similar between Tienam^®^ and Sun^®^, whereas D90 was markedly larger in Tienam^®^ than Sun^®^, with limited solubility in Visipaque^®^. In vivo, Tienam^®^ embolization led to variable reductions in neovascularization, ranging from complete to absent response (mean score decrease: 1.3 vs. 0.2 in controls, *p* = 0.07). Microbiological analysis revealed alterations in the rectal flora of all animals, with emergence of intrinsically carbapenem-resistant environmental species in four pigs, without evidence of acquired resistance. **Conclusions:** Imipenem–cilastatin demonstrates technical feasibility as a musculoskeletal embolic agent, but its efficacy remains heterogeneous, likely because of crystal irregularity and a broad size distribution. Although the procedure was clinically tolerated, the observed microbiota changes raise concerns regarding long-term safety and broader clinical adoption. Future research should focus on calibrated formulations that preserve embolic efficacy while improving reproducibility and minimizing off-target microbiological impact.

## 1. Introduction

Musculoskeletal disorders, including osteoarthritis and tendinopathies, are among the most prevalent chronic conditions worldwide, affecting more than 1.7 billion individuals globally [1]. These conditions are associated with persistent pain and functional impairment, representing a major public health concern with significant socioeconomic impact. Despite advances in medical and surgical management [2,3], a subset of patients continues to experience refractory pain [4]. For instance, up to 20% of patients report unexplained pain following total knee arthroplasty [5,6].

Recent insights into the pathophysiology of chronic musculoskeletal pain highlight the role of pathological neovascularization and the associated ingrowth of non-myelinated sensory nerve fibers [7,8,9]. These neurovascular structures are thought to perpetuate a vicious cycle of inflammation, neoangiogenesis, nociceptive signaling, and chronic pain. In tendinopathy, enthesopathy, and osteoarthritis, abnormal vascular proliferation is no longer regarded as a simple by-product of tissue remodeling but rather as an active contributor to pain persistence. Conventional imaging modalities, including Doppler ultrasound and angiography, highlight areas of hyperemia and vascular blush in symptomatic regions, particularly around tendons. These techniques can also identify abnormal hypervascular territories around entheses and synovium, thereby providing a mechanistic rationale for vascular-targeted therapies.

Interventions targeting pathological neovessels have emerged as a promising therapeutic alternative. Early studies reported analgesic benefits following the percutaneous injection of sclerosing agents into areas of neovascularization [10,11,12,13]. More recently, selective transarterial embolization (TAE) has shown encouraging results in multiple chronic pain syndromes, including shoulder, elbow, phantom limb and post-arthroplasty knee pain, for example [5,14,15,16,17,18,19]. Over the last decade, musculoskeletal embolization has also been reported in a growing range of refractory chronic pain conditions, including adhesive capsulitis, plantar fasciitis, and chronic low back pain, suggesting that the concept of vascular-targeted pain modulation is increasingly being explored across anatomical territories and clinical scenarios [14,15].

This expanding literature has progressively been consolidated by evidence syntheses. A systematic review dedicated to chronic musculoskeletal pain concluded that transarterial embolization appears to be a safe and effective treatment for selected forms of refractory musculoskeletal pain, while emphasizing the need for randomized placebo-controlled studies [14,16]. In refractory tendinopathy specifically, a recent systematic review and meta-analysis including 97 embolization procedures in 74 patients showed substantial short-term pain reduction from the first post-procedural day to 6 months, supporting the consistency of the analgesic signal observed across anatomical sites [15].

In knee osteoarthritis, where the evidence base is currently the most developed, systematic review and meta-analytic data have also shown durable improvements in pain and function after genicular artery embolization, with an overall favorable safety profile [17,18]. Recent clinical data further illustrate both the potential and the remaining uncertainties surrounding musculoskeletal embolization. In a large prospective single-center observational study including 194 patients with knee osteoarthritis, genicular artery embolization using rapidly resorbable gelatin-based microspheres was associated with substantial and sustained improvements in pain and function up to 12 months, with a favorable safety profile [19]. However, these encouraging uncontrolled results contrast with those of randomized sham-controlled trials. In a recent study of 58 patients with mild-to-moderate knee osteoarthritis, both genicular artery embolization and sham treatment resulted in sustained pain improvement at 12 months, with no significant between-group difference and no significant reduction in MRI-assessed synovitis [20]. This discrepancy suggests that the clinical response to embolization is likely multifactorial and that the respective contributions of vascular occlusion, patient selection, local vascular phenotype, embolic agent characteristics, and procedural or placebo effects remain incompletely understood.

From a personalized medicine perspective, musculoskeletal embolization is not intended as a uniform treatment for all painful tendinopathies [21]. Rather, it is designed for a selected subgroup of patients characterized by persistent symptoms despite conservative management, imaging and angiographic evidence of pathological neovascularization, and a clinical context in which a minimally invasive tissue-sparing approach may be preferable to surgery. In that framework, the embolic agent itself becomes a determinant of treatment personalization because its size distribution, solubility, and distal penetration condition both efficacy and safety at the individual lesion level.

Within this evolving field, imipenem–cilastatin, a third-generation carbapenem antibiotic co-formulated with cilastatin, has occupied a particularly important place and has been used off-label as an embolic agent in several studies [22]. Unlike calibrated permanent embolic agents, imipenem–cilastatin has been widely adopted in early musculoskeletal embolization studies because of its transient embolic effect, its ability to reach abnormal hyperemic microvascular territories, and the perception that this short-lived occlusion may reduce the risk of prolonged ischemia in non-target tissues [23]. The seminal work by Okuno et al. established the feasibility of transcatheter arterial embolization using imipenem–cilastatin sodium for tendinopathy and enthesopathy refractory to nonsurgical management [22]. Since then, this agent has been used in several musculoskeletal indications and has become one of the reference embolic materials in this field, particularly in exploratory and early clinical studies [15,16,23].

Although early clinical outcomes are promising, the embolic behavior, reproducibility, and safety implications of this approach remain insufficiently characterized [24]. A better understanding of imipenem–cilastatin physicochemical behavior is essential to move from empirical use toward a more reproducible phenotype-adapted embolization strategy [25,26]. For an innovative IR technique to be credibly integrated into personalized treatment pathways, a better understanding of embolization performance and off-target effects is required. Our study was designed to address this gap by combining in vitro physicochemical analyses with an in vivo assessment of embolization efficacy and safety in a porcine model of tendon neovascularization.

The collagenase-induced patellar tendinopathy model in the pig was selected over other candidate models because it reproducibly generates a localized quantifiable neovascular network at a caliber (tens to hundreds of micrometers) comparable to the pathological neovessels targeted clinically in human tendinopathy, is angiographically accessible via the genicular arteries with a technique directly transposable to human interventional practice, and has been previously validated and used as a training model for musculoskeletal embolization [27]. Its bilateral paired-limb design further allowed each animal to serve as its own internal control, maximizing statistical efficiency for a given number of animals in line with the 3Rs principle—an important consideration for evaluating a non-calibrated, off-label embolic agent for the first time in this controlled reproducible fashion.

## 2. Materials and Methods

### 2.1. Ethics Approval

All procedures were conducted in compliance with the EU Directive 2010/63/EU for animal experiments and institutional guidelines for animal research. Approval was obtained from the Animal Experimentation Ethics Committee (CEEA n° 036), and the project was authorized by the French Ministry of Higher Education, Research and Innovation under APAFIS authorization #33441-2021101214375516 (version 9, approved 29 March 2023).

### 2.2. In Vitro Analysis

Physico-chemical characterization of imipenem–cilastatin powders was performed on two commercial formulations, Tienam^®^ (Merck Sharp & Dohme, Hoddesdon, UK) and Sun^®^ (Sun Pharmaceutical Industries Ltd., Mumbai, India). Particle morphology was examined by scanning electron microscopy (SEM) (S-4800, Hitachi, Tokyo, Japan) and optical microscopy using a Keyence VHX-7000 3D microscope (Osaka, Japan). Particle size distribution was measured by dry laser diffraction granulometry (Mastersizer S 2.18, Malvern Instruments Ltd., Malvern, UK), with a jet pressure of 1.2 bar used for deagglomeration. The mean particle diameter (D [4,3]) and size distribution were calculated using Mastersizer S 2.18 software in polydisperse mode. Approximately 1 g of each powder was analyzed, and each measurement was performed at least in triplicate. The solid state of imipenem and cilastatin was further characterized by X-ray powder diffraction (XRPD) using a Bruker D8 Advance diffractometer (Karlsruhe, German) y with Cu Kα1 radiation (λ = 1.5406 Å, 40 kV, 40 mA), over an angular range of 2–60° 2θ with a step size of 0.02° and a counting time of 0.1 s per step.

Solubility tests were performed using 10 mL iodinated contrast media (Visipaque^®^ 320 mg/mL, GE Healthcare, Little Chalfont, UK) with separate testing of imipenem (European Pharmacopoeia (EP) Reference Standard, I0090000; Imipenem CRS. European Directorate for the Quality of Medicines & HealthCare (EDQM): Strasbourg, France, 2023) and cilastatin (European Pharmacopoeia (EP) Reference Standard, C2170000; Cilastatin Sodium CRS. European Directorate for the Quality of Medicines & HealthCare (EDQM): Strasbourg, France, 2023) solubility. Solubility tests were also performed with samples of imipenem–cilastatin from two manufacturers (Merck Sharp & Dohme, Hoddesdon, UK and Sun Pharmaceutical Industries Ltd., Mumbai, India).

### 2.3. Animal Model

To study neovessels and the embolizing effect of imipenem–cilastatin in vivo, we used the porcine model of collagenase-induced patellar tendinopathy, developed by J. Ghelfi et al. [27].

This pilot study was conducted without formal sample size calculation, reflecting its exploratory hypothesis-generating nature; it was designed to provide preliminary feasibility, efficacy, and safety data to inform the sample size of a subsequent adequately powered confirmatory study, consistent with common practice for early-stage preclinical proof-of-concept studies and with the 3Rs principle of minimizing animal use. The animals were conventional commercial slaughter pigs, with no known genetic modifications, of standard health and immune status, who had not undergone any previous experimental procedures.

Six 3 months pigs (3 males, 3 females) were included. Animals were housed in groups of three, with a five-day acclimatization period prior to the procedure. As each animal served as its own control, no additional animal isolation was required.

Neovascularization in the patellar tendon was induced by injecting 25 mg of collagenase (Sigma-Aldrich, St Louis, MO, USA) under ultrasound guidance.

At D7 after induction, under general anesthesia, diagnostic angiography was performed followed by transarterial embolization of the genicular arteries using either 0.5 g of a suspension of imipenem–cilastatin (Tienam^®^) mixed with 10 mL of Visipaque^®^ or Visipaque^®^ alone. Each pig underwent bilateral embolization: one randomly selected limb was treated with imipenem–cilastatin, while the contralateral limb received contrast media only, serving as an internal control. For each animal, the side allocated to embolization was determined by simple randomization: an independent operator (the animal facility zootechnician), not otherwise involved in the injection procedure or in the angiographic scoring, manually drew lots to designate the treated and control limbs (no computer-generated randomization sequence was used).

At D14, a control arteriography was performed under the same conditions as the previous phase. This second angiographic evaluation aimed to assess post-embolization changes in neovascularization, using a validated 4-point Likert scale [27].

In addition, arterial anastomoses and early venous returns were assessed using a 3-point Likert scale (0 = absent, 1 = present, 2 = marked) [27]. Two radiologists (one junior, one senior) independently evaluated the images. Both were blinded to the treatment allocation. To minimize the recall bias, image analysis was conducted after a three-week washout period. Discrepancies were resolved by consensus.

Tienam^®^ was used for the in vivo phase because it was the imipenem–cilastatin formulation readily available through the hospital pharmacy at Nîmes University Hospital, where the animal procedures were performed. Sun^®^, a second commercial formulation, was included only in the in vitro comparison to benchmark particle characteristics across two different manufacturers; it was obtained from Montpellier University Hospital, where it was stocked, since it was not available through the Nîmes University Hospital pharmacy, which precluded its use in vivo.

### 2.4. Safety

Clinical follow-up included the daily assessment of appetite, activity and locomotion, using a scale routinely used by the team of zootechnicians at the experimental research laboratory. Environmental enrichment was provided through a covered resting area with bedding, allowing group resting and suspended chewable toys. Animal caretakers were blinded to treatment allocation during clinical evaluations.

As imipenem is a third-generation antibiotic, it exerts selective pressure on microorganisms present in the body. To assess the potential emergence of carbapenem-resistant bacteria, rectal swabs were collected from anesthetized pigs on the day of embolization, before intra-arterial injection of imipenem–cilastatin (D7), and on the final day before control arteriography (D14). The sample was inoculated onto selective agar plates for carbapenem-resistant Gram-negative bacilli, chromID^®^ OXA-48 and chromID^®^ CARBA (bioMérieux, Marcy l’Etoile, France). After 18–24 h of incubation in aerobic conditions at 35 ± 2 °C, the bacterial colonies were semi-quantified, and the species were identified using MALDI-TOF mass spectrometry (Vitek MS, bioMérieux, Marcy-l’Étoile, France).

### 2.5. Statistical Analysis

Statistical analysis was performed using R Studio software (version 4.5.2, R Foundation for Statistical Computing, Vienna, Austria). Quantitative variables were described by mean ± standard deviation and median with 10th and 90th percentiles (D10–D90). Given the small sample size, the independence of the groups and the non-normality observed, comparisons were made using a non-parametric Mann–Whitney test. The significance threshold was set at α = 0.05.

The primary exploratory endpoint was the change in angiographic neovascularization score between D7 and D14. Secondary exploratory endpoints were changes in arterial anastomoses and early venous return scores, together with descriptive safety outcomes, including clinical tolerance and rectal microbiological findings.

## 3. Results

### 3.1. Physicochemical Properties

Optical (Figure 1A) and SEM imaging (Figure 1B) realized on commercial Tienam^®^ revealed heterogeneous morphologies with irregular often agglomerated crystal shapes. Several crystalline morphologies were observed, with some particles exhibiting complex, often polygonal shapes, while others appeared more rounded. Polygonal forms represented the predominant morphology in the analyzed sample.

Laser diffraction analysis of crystals Tienam^®^ (Figure 2) showed a median diameter (D50) of 30.2 ± 0.9 µm. The size distribution is heterogenous, with a narrow peak centered around 50 µm but numerous outliers exceeding 100 µm. These findings highlight the coexistence of fine particles and larger aggregates within the same sample.

The size distribution of the two commercial formulations, Tienam^®^ and Sun^®^, was also compared (Figure 3). It is interesting to note that while the D50 values were similar (30.2 ± 0.9 µm and 26.96 ± 0.7 µm, respectively), indicating comparable median particle sizes, marked differences were observed in the distribution of larger particles. It was found that D90 was substantially higher for Tienam^®^ (341.2 ± 27.4 µm) than for Sun^®^ (125.6 ± 24.7 µm), revealing a larger proportion of large crystals in the former. This finding was further supported by the D [4,3] value, which represents the volume-weighted mean diameter and is particularly sensitive to the presence of larger particles. Consistently, D [4,3] was also higher for Tienam^®^ than for Sun^®^ (105.6 ± 8.3 and 59.8 ± 12.5 µm, respectively), confirming that the Tienam^®^ formulation contained a larger contribution of large particles to the overall sample volume.

The solubility of commercial imipenem–cilastatin was evaluated in the iodinated contrast medium Visipaque^®^. The results indicated that imipenem–cilastatin crystals were generally poorly soluble under the experimental conditions, leading to the formation of persistent aggregates, some exceeding 100 µm in diameter (Figure 4).

Interestingly, the solubility tests performed on the individual components showed that cilastatin dissolved rapidly and completely in the contrast medium, whereas imipenem exhibited very low solubility. Residual imipenem particles were still observed after 45 min, supporting the hypothesis that the embolic effect observed is primarily attributable to imipenem. This was further supported by X-ray diffraction (XRD) analysis (Figure 5). The diffraction pattern of the crystals remaining after 45 min in suspension in the contrast agent closely matched that of imipenem alone, rather than cilastatin or the initial commercial imipenem–cilastatin formulation.

### 3.2. Clinical and Angiographic Finding

All animals and all planned analyses were included in the results, with no exclusions or discarded data.

All animals tolerated the embolization procedure without clinical complications. Behavioral monitoring revealed a transient decrease in clinical scores following collagenase injection, followed by a progressive return to baseline during the second postoperative week. One temporary livedo was observed after arterial injection but resolved spontaneously without skin necrosis.

Initial angiographic evaluation performed prior to embolization showed comparable neovascularization scores between groups, with a mean score of 2.7 (±0.5) in the imipenem–cilastatin group and 2.8 (±0.4) in the controls (Table 1). On follow-up angiography at day 14, a reduction in neovascularization was observed in both groups, with a mean decrease of 1.3 points (±1.37) in the imipenem–cilastatin group versus 0.17 (±0.4) in the control group. The difference in score reduction between the treatment and control groups was not statistically significant (*p* = 0.07; Hodges–Lehmann estimated median difference 1.0 point, 95% CI 0.0 to 3.0). Within the imipenem–cilastatin group, the outcomes were heterogenous: two limbs exhibited complete resolution of visible angiographic hypervascularity (grade 0), two exhibited partial reduction, while two animals exhibited no detectable angiographic change. Comparable heterogeneity was noted in the evolution of arterial anastomoses and early venous drainage patterns (Figure 6).

### 3.3. Microbiological Evaluation

Rectal swabs collected before and after intra-arterial administration of low-dose imipenem–cilastatin revealed qualitative changes in the intestinal flora (Table 2).

Before embolization (D7), all pigs presented a mixed flora mainly composed of environmental bacteria (*Ochrobactrum anthropi*) and yeasts (*Candida catenulata*, *Trichosporon asahii*). After treatment (D14), five out of six animals showed the emergence of new environmental Gram-negative bacilli. Specifically, *Pseudomonas putida* appeared in P1, *Brevundimonas diminuta* and *Achromobacter xylosoxidans* in P2, *Acinetobacter johnsonii* in P4, and Stenotrophomonas maltophilia together with *A. johnsonii* in P5 and P6.

Four animals showed species naturally resistant to certain β-lactams, including ertapenem (and in the case of *S. maltophilia*, imipenem), but no acquired resistance phenotype or emergence of carbapenemase-producing strains was detected.

## 4. Discussion

To strengthen the translational relevance of the study, the experimental design intentionally combined complementary levels of evaluation: (i) bench characterization of particle morphology, distribution, and solubility; (ii) in vivo assessment of angiographic response in a standardized neovascularization model; and (iii) exploratory microbiological safety monitoring. This multilevel approach was chosen because personalization in embolization depends not only on the target-vessel occlusion but also on predicting how a given formulation behaves in vivo and whether its antibiotic nature may have off-target biological consequences.

### 4.1. Primary Efficacy and Angiographic Variability

Our findings support the translational potential of imipenem–cilastatin in a porcine model of musculoskeletal neovascularization. The observed reduction in vascular score, although not statistically significant, is directionally in line with clinical improvements previously reported in tendinopathies treated by imipenem embolization, although this non-significant preclinical signal should not be equated with proven clinical efficacy. However, the embolization efficacy was variable, highlighting a key challenge for the broader adoption and standardization of this innovative IR technique. Beyond efficacy itself, the broad crystal size distribution observed in our study also appears to be a critical issue, as such heterogeneity may directly influence embolic behavior, distal vessel penetration, and the consistency of the angiographic and biological response.

At the individual level, the angiographic trajectories underline this variability: some treated animals showed a marked suppression of neovessels, whereas others had only a partial or absent response despite the same procedural framework. Such variability may be related not only to the heterogeneous crystal size distribution but also to differences in local vascular architecture. Indeed, the embolic behavior of the suspension is likely to vary according to the vessel caliber, with larger particles causing more proximal occlusion and smaller particles achieving more distal penetration. Moreover, arterial anastomoses and venous outflow may influence particle distribution and clearance, thereby limiting effective target embolization in some cases. Taken together, these factors may contribute to the heterogeneity of the angiographic response observed despite an apparently standardized technique.

### 4.2. Comparative Physicochemical Mechanics

This heterogeneity may be explained by the physicochemical characteristics of the agent, including the variability in particle morphology, size distribution, and solubility.

Our findings are consistent overall with those of Nakamura et al., who also reported that imipenem–cilastatin particles are predominantly polygonal and display a transient embolic profile in vitro [24]. However, their analysis relied mainly on manually selected microscopic fields, with the particle size assessed by measuring the long axis of visually isolated particles and particle shape classified by visual review. In their study, 244 particles were measured for size and 109 for shape, after exclusion of suspected aggregates, which provides useful descriptive data but may not fully capture the entire size distribution, especially its coarser fraction. Another important difference is that Nakamura et al. evaluated a Merck formulation, whereas in our study we analyzed two different commercial formulations, Tienam^®^ and Sun^®^. This broader comparative approach further highlights that the crystal size distribution may vary according to the manufacturer, reinforcing the idea that imipenem–cilastatin cannot be considered a fully homogeneous embolic material across brands. By contrast, our methodology was more exhaustive and more standardized, as it combined microscopy with automated particle sizing approaches and comparative analysis between formulations, allowing a more robust characterization of particle heterogeneity and size distribution.

Larger or irregular particles may result in proximal arterial occlusion, thereby preventing effective distal embolization. In this context, both the presence of larger crystals and the marked heterogeneity in particle size distribution may represent major determinants of the variability in embolization efficacy observed in our study. In contrast, more uniform and optimally sized crystals are likely to penetrate further into the microvasculature and achieve more consistent vessel occlusion. Our solubility analyses also indicate that only the imipenem component acts as the actual embolic agent, as cilastatin dissolves rapidly in contrast media. Taken together, these in vitro findings indicate that the commercially available formulations tested do not behave as calibrated embolic particles. Instead, they generate a broad and partially unstable crystal population, which is relevant for personalized embolization because distal penetration is likely to vary according to the local vascular bed and to the formulation used.

Interestingly, these challenges mirror those historically encountered with the use of polyvinyl alcohol (PVA) particles. Developed in the 1970s as embolic agents, PVA particles have shown similarly inconsistent occlusion behavior. Bendszus et al. demonstrated that PVA often causes proximal obstruction compared to more distally acting trisacryl gelatin microspheres, which are homogeneous and size-calibrated, thereby reducing surgical blood loss in preoperative meningioma embolization [28]. Other studies have revealed that PVA particles range from 10 µm to over 1500 µm and tend to form aggregates that compromise their embolic precision [25,26]. Moreover, PVA particles have been reported to increase in size upon suspension in contrast agents. This mirrors our own findings with imipenem-cilastatin crystals and may partly explain the incomplete embolization observed in some cases. Similar concerns have been raised by Woodhams et al., who reported a 45% rebleeding rate in patients treated with imipenem–cilastatin for gastrointestinal tumor hemorrhage [29]. Our results support the idea that crystal homogeneity, size control, and solubility are critical parameters in the design of ideal embolic agents for musculoskeletal applications.

### 4.3. Microbiological Safety and Ecological Implications

While no carbapenemase-producing or clinically resistant strains emerged, the consistent replacement of commensal flora by intrinsically resistant environmental species suggests an ecological disturbance induced by intra-arterial exposure to imipenem–cilastatin. Such bacteria—Stenotrophomonas maltophilia, *Achromobacter xylosoxidans*, and *Acinetobacter johnsonii*—are well known for their environmental persistence and intrinsic resistance mechanisms. These findings raise concerns about the potential consequences of repeated or large-scale use of intra-arterial carbapenems, which might inadvertently select and disseminate environmental microorganisms capable of harboring resistance determinants. Even in the absence of acquired resistance, the observed ecological shift underscores the need for the cautious and judicious application of local antibiotic therapies.

### 4.4. Study Limitations

This study has several limitations. First, the sample size was limited, reducing the statistical power and likely contributing to the absence of a significant difference between treatment and control groups despite visible inter-individual angiographic variations. This limited power also restricts the interpretation of negative findings and makes it difficult to distinguish a true lack of efficacy from a heterogeneous treatment response. Second, although the porcine model reliably reproduces patellar tendon neovascularization and allows standardized angiographic follow-up, it remains a preclinical model that does not fully capture the clinical complexity of chronic tendinopathy in humans. In particular, the short experimental window precludes meaningful correlation with symptoms, function, or pain relief, which are central endpoints in musculoskeletal embolization. Third, the angiographic assessment of neovascularization has inherent technical and interpretative limitations. The degree of vessel opacification may vary according to the catheter position, injection rate, and contrast medium dynamics, all of which can influence the apparent extent of neovascular blush and the perceived embolization endpoint. In addition, grading neovascularization remains partly subjective and may be affected by interobserver variability. The development of complementary quantitative imaging approaches, including MRI-based or perfusion-based vascular assessment, could help improve standardization and reduce observer-dependent bias [30]. Fourth, our study was not designed to define the optimal embolization endpoint or to compare different injection strategies. Because imipenem/cilastatin is a transient and non-calibrated embolic agent, procedural reproducibility may also have been affected by the variability in crystal size distribution, suspension characteristics, distal penetration, and washout through arterial anastomoses or venous outflow. These factors may have contributed substantially to the heterogeneity of angiographic response observed despite an apparently standardized procedural framework. Fifth, the microbiota findings should be interpreted with caution. Although we attempted to control the environmental conditions through a five-day acclimatization period, changes in gut microbiota may still have been influenced by housing conditions, diet, or other non-procedural factors. Sixth, the microbiological comparison lacked a parallel unexposed control group housed under identical conditions; because the animals shared housing, bedding and enrichment items, facility-acquired environmental colonization or cross-contamination cannot be fully excluded as a contributor to the observed flora shift, even though each animal served as its own before/after (D7 vs. D14) comparison. Seventh, using the contralateral limb as an internal control assumes negligible systemic redistribution of imipenem–cilastatin to the non-embolized side; as serum pharmacokinetics were not measured, a systemic contribution to vascular dynamics in the control limb cannot be entirely ruled out. Finally, given the exploratory nature of this pilot study and the small number of animals, angiographic scores were assigned by blinded consensus between the two readers rather than by two fully independent separately archived readings; formal inter-observer agreement (e.g., Cohen’s kappa) between the two readers was therefore not quantified, and this should be incorporated, alongside fully independent scoring, in future larger-scale studies to further strengthen the reproducibility of the angiographic endpoint. Larger studies with longer follow-up, stricter biological standardization, and comparative evaluation against other embolic agents will be needed to clarify both the efficacy and safety profile of imipenem–cilastatin in musculoskeletal embolization.

## 5. Conclusions

Imipenem–cilastatin demonstrates technical feasibility as an embolic agent for musculoskeletal applications in interventional radiology. However, its variable efficacy appears linked to irregular crystal morphology, broad size distribution, and limited solubility of imipenem in iodinated contrast media, all of which may compromise consistent distal vessel occlusion. In parallel, although no acquired carbapenem-resistant bacteria emerged, the observed shift toward intrinsically resistant environmental species suggests that even low-dose intra-arterial imipenem–cilastatin may alter the microbiological ecosystem and should therefore be used with caution, especially if repeated treatments or wider clinical implementation are considered. Future research should aim to develop these areas.

## Figures and Tables

**Figure 1:**
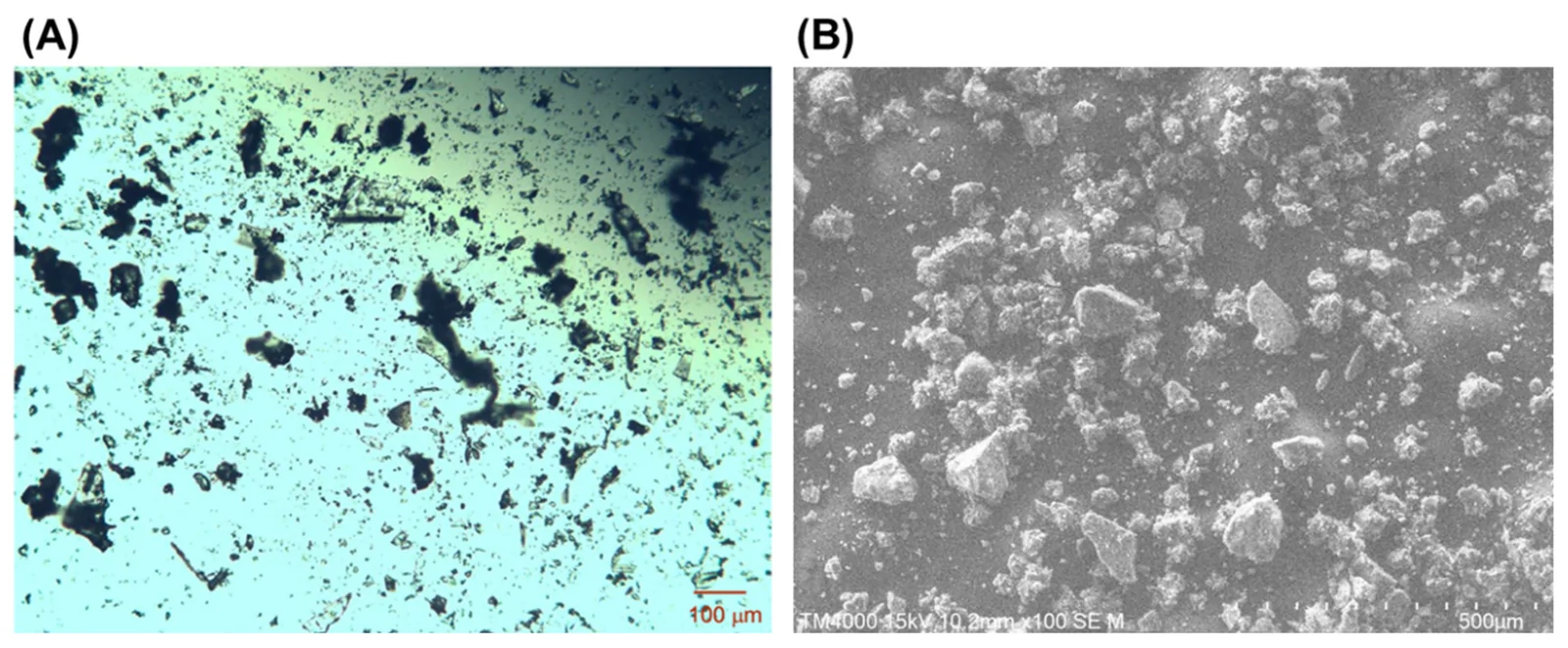
(**A**) Optical microscopy image of commercial imipenem–cilastatin crystals (Tienam^®^); (**B**) Scanning electron microscopy (SEM) of imipenem–cilastatin (Tienam^®^) at ×100 magnification.

**Figure 2:**
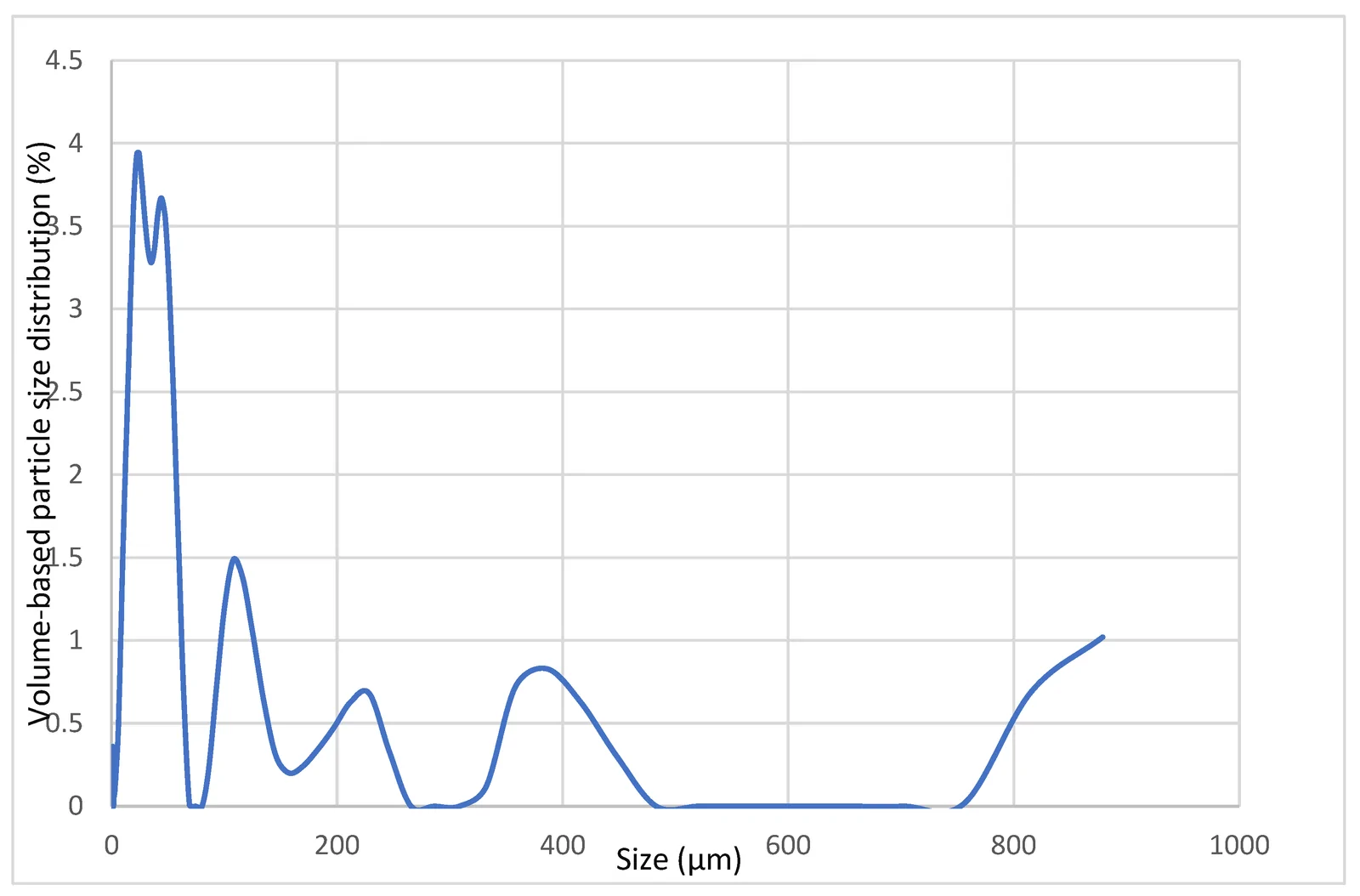
Volume-based particle size distribution of imipenem–cilastatin crystals Tienam^®^ measured by laser diffraction.

**Figure 3:**
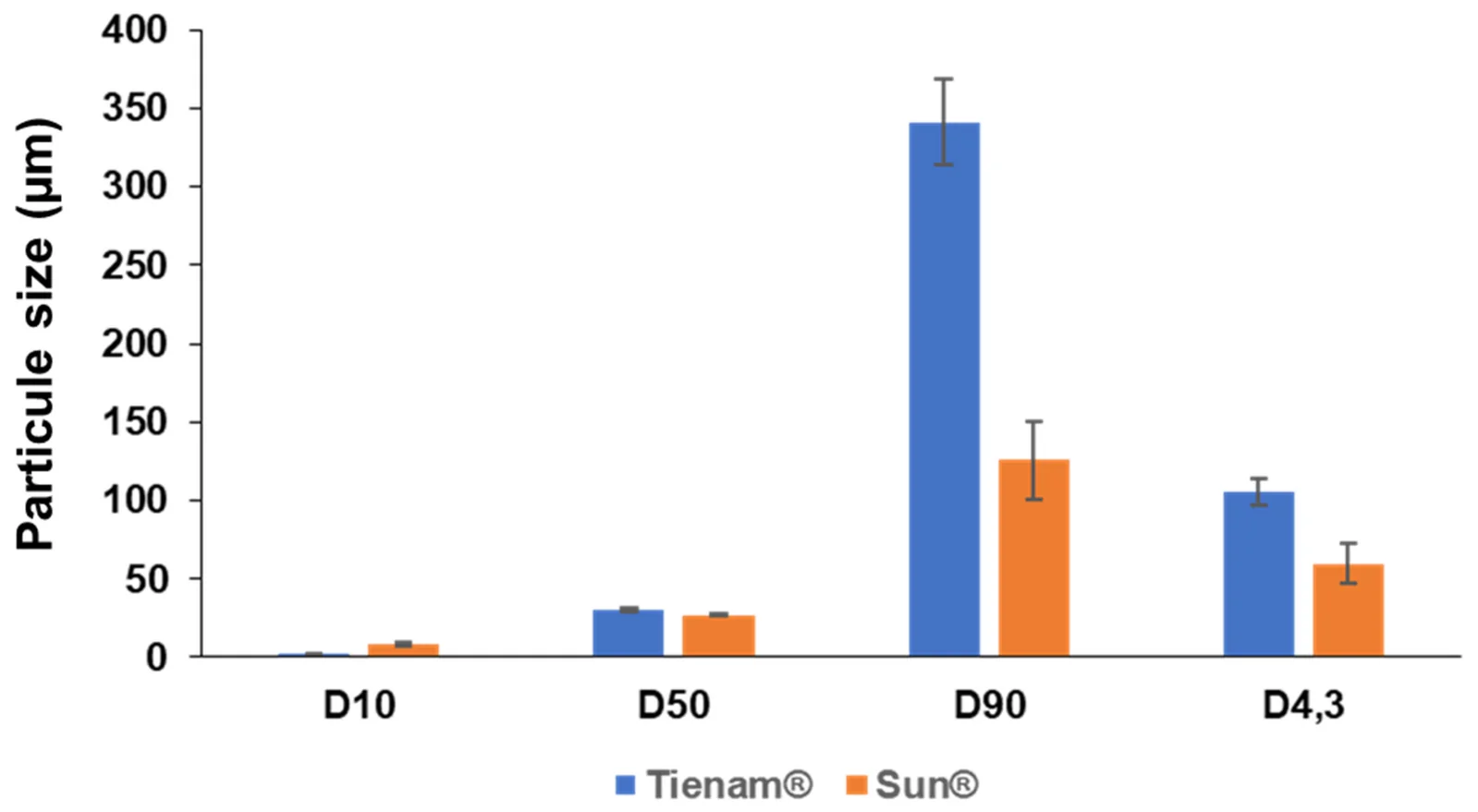
Comparison of particle size distribution between Tienam^®^ and Sun^®^ formulations of imipenem–cilastatin.

**Figure 4:**
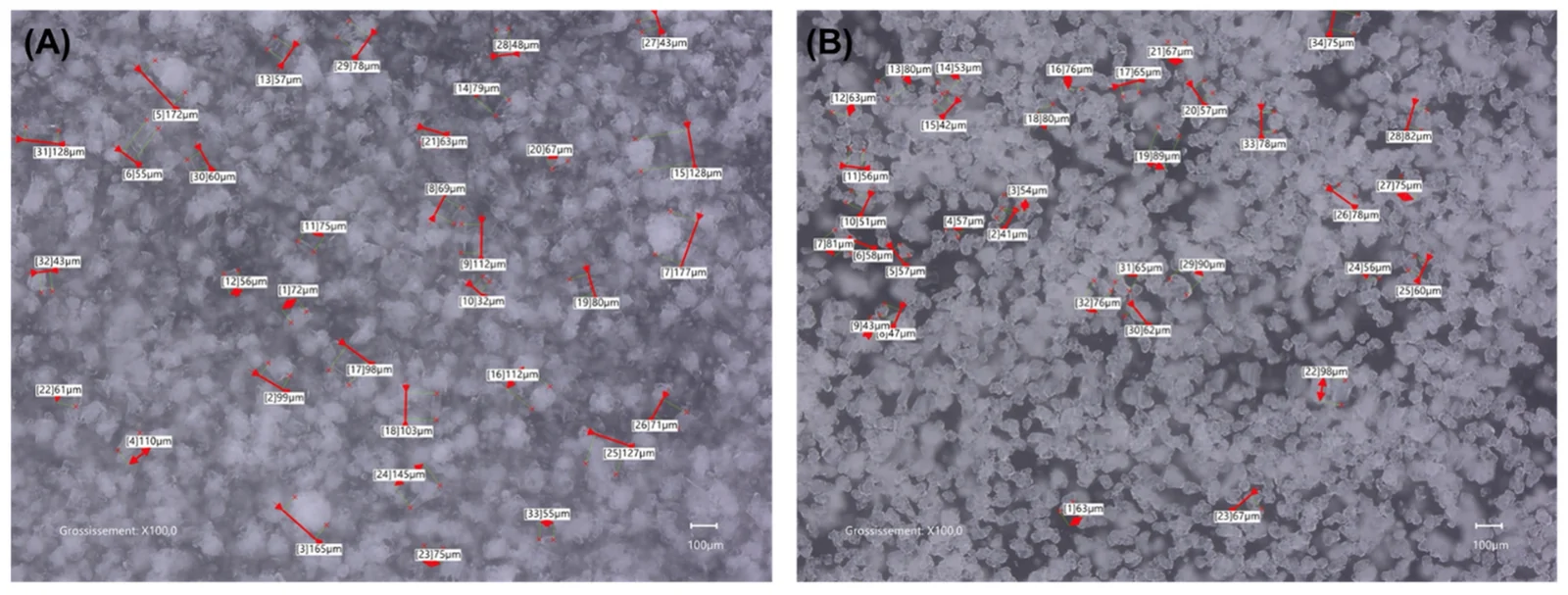
Solubility assessment of imipenem–cilastatin at 45 min; (**A**) Tienam ^®^ (**B**) Sun ^®^.

**Figure 5:**
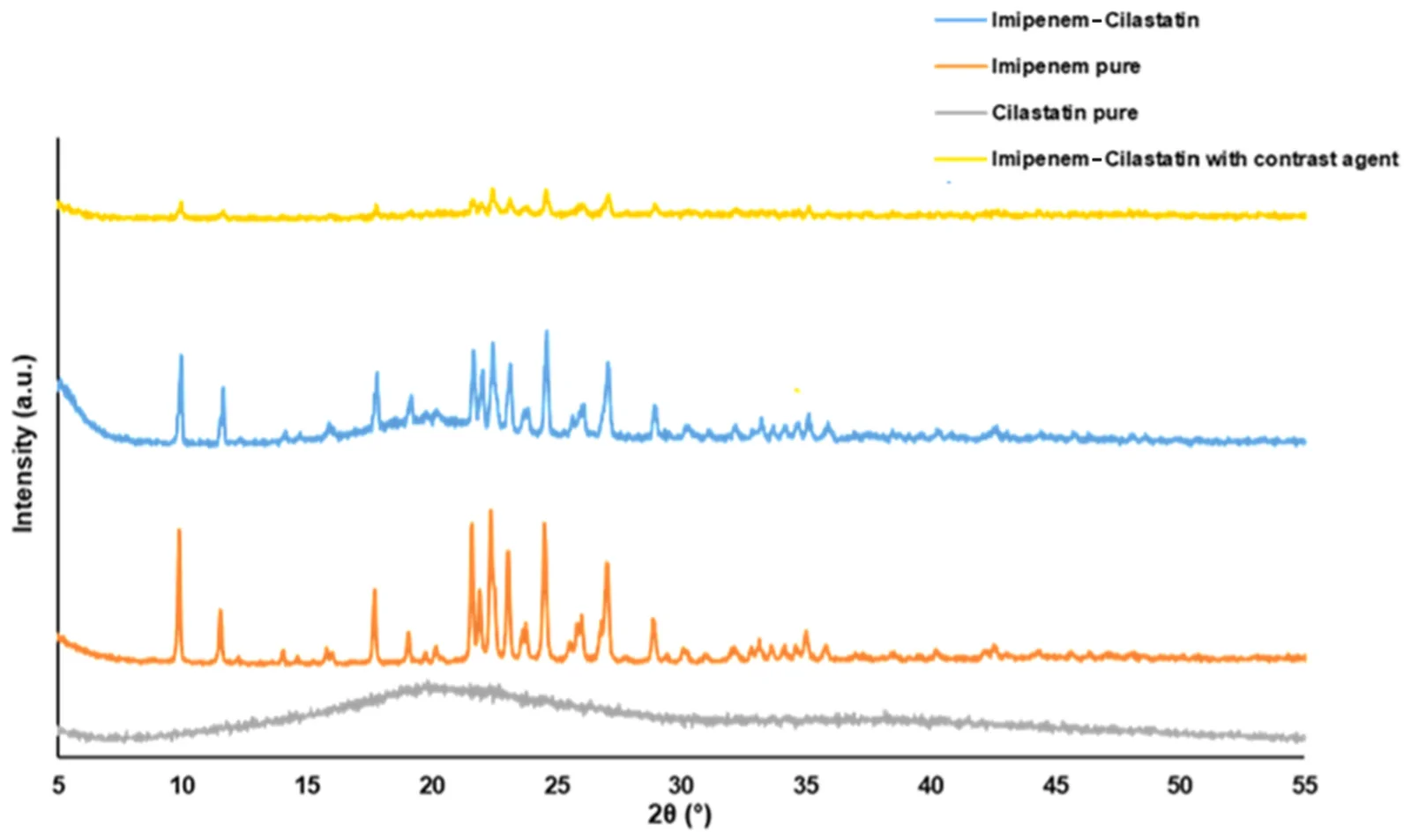
XRPD patterns of cilastatin pure, imipenem pure, commercial imipenem–cilastatin and commercial imipenem–cilastatin after 45 min in the contrast media Visipaque^®^. Similar results were obtained for Tienam^®^ and Sun^®^.

**Figure 6:**
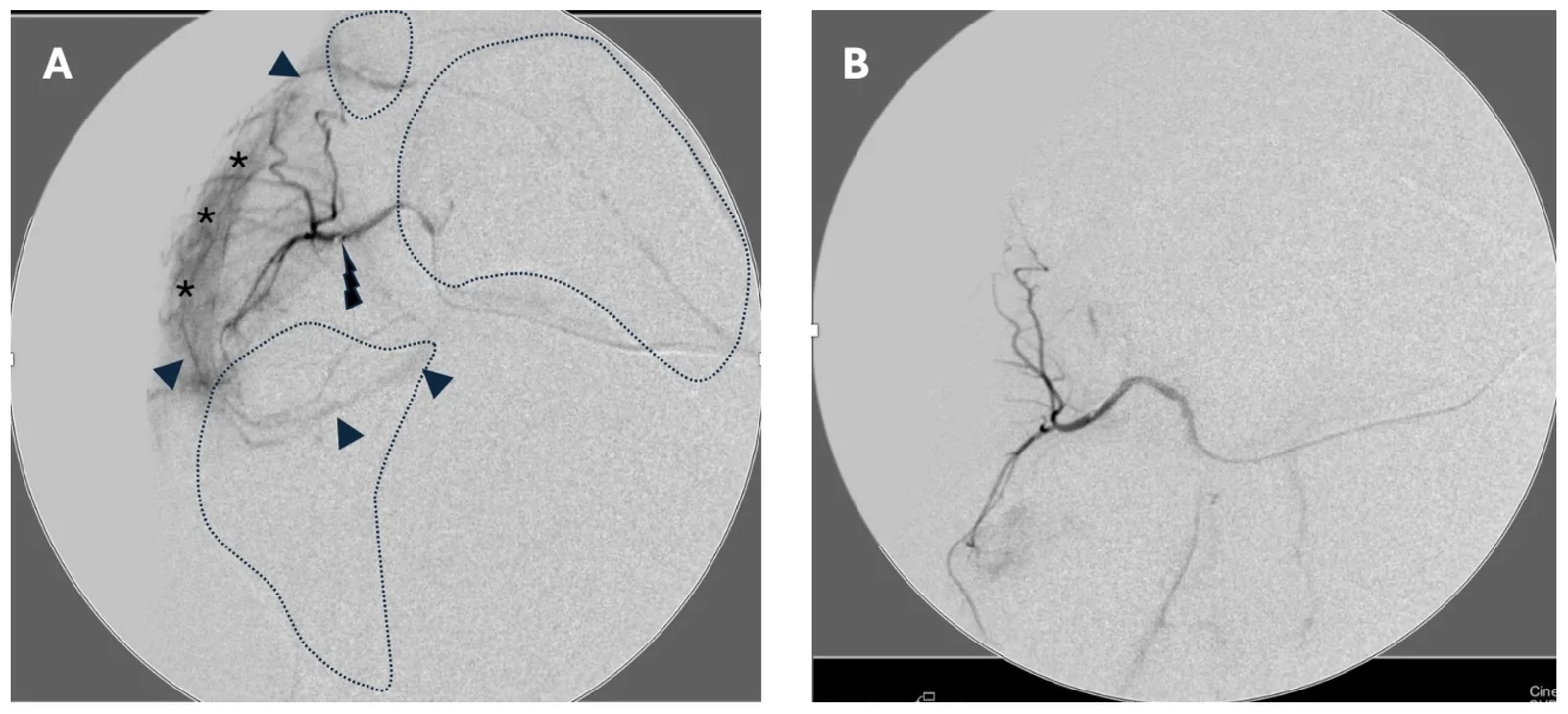
Selective digital subtraction angiography of the middle genicular artery before embolization (**A**) and angiographic follow up at 1 week (**B**). Selective digital subtraction angiography performed through a microcatheter positioned in the middle genicular artery (black lightning symbol). (**A**) Day 7 angiogram showing grade 3 neovascularization within the patellar tendon (black asterisks), associated with arterial abnormalities including arterial anastomoses and early venous return (black arrowheads). (**B**) Follow-up angiogram obtained 1 week later (Day 14), showing disappearance (grade 0) of the abnormal neovessels. For anatomical orientation, the femur, patella, and tibia are outlined with black dotted lines.

**Table 1:** Evolution of neovascularization, arterial anastomoses, and early venous return scores.

Imipenem-Cilastatin Group							
Pig ID (P)	Angiographic Score D7	Angiographic Score D14	Change	Arterial Anastomoses D7	Arterial Anastomoses D14	Early Venous Return D7	Early Venous Return D14
P 1	3	0	3	2	0	0	0
P 2	3	3	0	1	1	1	0
P 3	2	1	1	2	1	0	0
P 4	3	0	3	0	0	2	0
P 5	3	3	0	2	2	0	0
P 6	2	1	1	2	0	2	1
**Control Group**							
**Pig ID (P)**	**Angiographic Score** **D7**	**Angiographic Score** **D14**	**Change**	**Arterial Anastomoses D7**	**Arterial Anastomoses D14**	**Early Venous Return** **D7**	**Early Venous Return** **D14**
P 1	2	1	1	1	1	2	2
P 2	3	3	0	2	2	1	1
P 3	3	3	0	2	2	0	0
P 4	3	3	0	1	1	0	0
P 5	3	3	0	2	2	1	0
P 6	3	3	0	2	2	0	0

**Table 2:** Microbiological evaluation.

Pig	Microorganisms Isolated After Rectal Screening on Selective Medium (chromID^®^ CARBA SMART, bioMérieux)	
	Before Injection of Imipenem–Cilastatin	D7 After Injection of Imipenem–Cilastatin
P 1	Few colonies of *Ochrobactrum anthropi*, numerous colonies of *Candida catenulata*	Few colonies of *Ochrobactrum anthropi* and *Pseudomonas putida*
P 2	Few colonies of *Ochrobactrum anthropi*, *Candida catenulata* and *Trichosporon asahii*	Numerous colonies of *Brevundimonas diminuta* and *Achromobacter xylosoxidans* (environmental Gram-negative bacilli naturally resistant to ertapenem)
P 3	Few colonies of *Ochrobactrum anthropi*, numerous colonies of *Candida catenulata*	Numerous colonies of *Ochrobactrum anthropi* and *Candida catenulata*
P 4	Few colonies of *Ochrobactrum anthropi*, *Candida catenulata* and *Trichosporon asahii*	Numerous colonies of *Acinetobacter johnsonii* (naturally resistant to ertapenem)
P 5	Few colonies of *Ochrobactrum anthropi*, *Candida catenulata* and *Trichosporon asahii*	Numerous colonies of Stenotrophomonas maltophilia and *Acinetobacter johnsonii* (environmental Gram-negative bacilli; *S. maltophilia* is naturally resistant to imipenem and *A. johnsonii* to ertapenem)
P 6	Few colonies of *Ochrobactrum anthropi*, *Candida catenulata* and *Trichosporon asahii*	Numerous colonies of Stenotrophomonas maltophilia and *Acinetobacter johnsonii* (environmental Gram-negative bacilli; *S. maltophilia* is naturally resistant to imipenem and *A. johnsonii* to ertapenem)

## Data Availability

The datasets used and analyzed during the study remain are available from the corresponding author on reasonable request.

## References

[1] Cieza A., Causey K., Kamenov K., Hanson S.W., Chatterji S., Vos T. (2020). Global estimates of the need for rehabilitation based on the Global Burden of Disease study 2019: A systematic analysis for the Global Burden of Disease Study 2019. Lancet.

[2] Millar N.L., Silbernagel K.G., Thorborg K., Kirwan P.D., Galatz L.M., Abrams G.D., Murrell G.A.C., McInnes I.B., Rodeo S.A. (2021). Tendinopathy. Nat. Rev. Dis. Prim..

[3] Chen P.-C., Wu K.-T., Chou W.-Y., Huang Y.-C., Wang L.-Y., Yang T.-H., Siu K.-K., Tu Y.-K. (2019). Comparative Effectiveness of Different Nonsurgical Treatments for Patellar Tendinopathy: A Systematic Review and Network Meta-analysis. Arthroscopy.

[4] Wylde V., Hewlett S., Learmonth I.D., Dieppe P. (2011). Persistent pain after joint replacement: Prevalence, sensory qualities, and postoperative determinants. Pain.

[5] Lewis G.N., Rice D.A., McNair P.J., Kluger M. (2015). Predictors of persistent pain after total knee arthroplasty: A systematic review and meta-analysis. Br. J. Anaesth..

[6] Chau Y., Roux C., Breuil V., Trojani C., Gonzalez J.-F., Amoretti N., Sédat J. (2020). Endovascular Occlusion of Neovascularization as a Treatment for Persistent Pain After Total Knee Arthroplasty. Cardiovasc. Interv. Radiol..

[7] Ashraf S., Wibberley H., Mapp P.I., Hill R., Wilson D., Walsh D.A. (2011). Increased vascular penetration and nerve growth in the meniscus: A potential source of pain in osteoarthritis. Ann. Rheum. Dis..

[8] Ohberg L., Lorentzon R., Alfredson H. (2001). Neovascularisation in Achilles tendons with painful tendinosis but not in normal tendons: An ultrasonographic investigation. Knee Surg. Sports Traumatol. Arthrosc..

[9] Xu Y., Bonar F., Murrell G.A.C. (2011). Neoinnervation in rotator cuff tendinopathy. Sports Med. Arthrosc. Rev..

[10] Zeisig E., Ohberg L., Alfredson H. (2006). Sclerosing polidocanol injections in chronic painful tennis elbow-promising results in a pilot study. Knee Surg. Sports Traumatol. Arthrosc..

[11] Alfredson H., Ohberg L. (2005). Neovascularisation in chronic painful patellar tendinosis—Promising results after sclerosing neovessels outside the tendon challenge the need for surgery. Knee Surg. Sports Traumatol. Arthrosc..

[12] Hoksrud A., Torgalsen T., Harstad H., Haugen S., Andersen T.E., Risberg M.A., Bahr R. (2012). Ultrasound-guided sclerosis of neovessels in patellar tendinopathy: A prospective study of 101 patients. Am. J. Sports Med..

[13] Ohberg L., Alfredson H. (2003). Sclerosing therapy in chronic Achilles tendon insertional pain-results of a pilot study. Knee Surg. Sports Traumatol. Arthrosc..

[14] Kishore S., Sheira D., Malin M.L., Trost D.W., Mandl L.A. (2022). Transarterial Embolization for the Treatment of Chronic Musculoskeletal Pain: A Systematic Review of Indications, Safety, and Efficacy. ACR Open Rheumatol..

[15] Epelboym Y., Glaser C., Lan Z., Mandell J.C., Burch E., Killoran T., Guermazi A. (2024). Transcatheter arterial tendinopathy embolization as a treatment for painful and refractory tendinopathy: A systematic review and meta-analysis. Skelet. Radiol..

[16] Hindsø L., Riis R.G.C., Hölmich P., Petersen M.M., Nielsen M.B., Lönn L., Taudorf M. (2021). Current Status of Trans-Arterial Embolization in Pain Management of Musculoskeletal Inflammatory Conditions—An Evidence-Based Review. Cardiovasc. Interv. Radiol..

[17] Torkian P., Golzarian J., Chalian M., Clayton A., Rahimi-Dehgolan S., Tabibian E., Talaie R. (2021). Osteoarthritis-Related Knee Pain Treated with Genicular Artery Embolization: A Systematic Review and Meta-analysis. Orthop. J. Sports Med..

[18] Chlorogiannis D.-D., Vasilopoulou A., Konstantinidis C.I., Pagona A.E., Filippiadis D.K. (2024). Knee pain improvement after genicular artery embolization for the management of knee osteoarthritis: An updated systematic review and meta-analysis of 21 studies. Radiologie.

[19] Fleckenstein F.N., David D., Garducci P., Maleitzke T., Oehme S., Savic L.J., Auer T.A., Gebauer B., Winkler T., Collettini F. (2026). Genicular Artery Embolization Using Rapidly Resorbable Gelatin-based Microspheres for Osteoarthritis-related Knee Pain. Radiology.

[20] van Zadelhoff T.A., van der Heijden R.A., Bierma-Zeinstra S.M.A., Bos P.K., Oei E.H.G. (2026). Long-term outcomes of genicular artery embolization for knee osteoarthritis: 12-month efficacy and secondary outcomes from a randomized sham-controlled clinical trial. Eur. Radiol..

[21] Padia S.A. (2025). How I Do It: Embolization for Joint Pain. Radiology.

[22] Okuno Y., Matsumura N., Oguro S. (2013). Transcatheter Arterial Embolization Using Imipenem/Cilastatin Sodium for Tendinopathy and Enthesopathy Refractory to Nonsurgical Management. J. Vasc. Interv. Radiol..

[23] Koucheki R., Dowling K.I., Patel N.R., Matsuura N., Mafeld S. (2021). Characteristics of Imipenem/Cilastatin: Considerations for Musculoskeletal Embolotherapy. J. Vasc. Interv. Radiol..

[24] Nakamura H., Yamamoto A., Fukunaga T., Watanabe H., Ito K., Higaki A., Kanki A., Fukukura Y., Tamada T. (2024). In vitro study of the embolic characteristics of imipenem/cilastatin particles. CVIR Endovasc..

[25] Ji J., Fang S., Chen M., Zheng L., Chen W., Zhao Z., Cheng Y. (2021). Precision interventional radiology. J. Interv. Med..

[26] Uldin H., Kanbour I., Patel A., Botchu R. (2024). Image-Guided Musculoskeletal Interventional Radiology in the Personalised Management of Musculoskeletal Tumours. J. Pers. Med..

[27] Gremen E., Ghelfi J., Bacle M., Frandon J. (2025). Proposal for training: The educational value of a musculoskeletal embolization patellar tendinopathy model. Diagn. Interv. Radiol..

[28] Bendszus M., Klein R., Burger R., Warmuth-Metz M., Hofmann E., Solymosi L. (2000). Efficacy of trisacryl gelatin microspheres versus polyvinyl alcohol particles in the preoperative embolization of meningiomas. AJNR Am. J. Neuroradiol..

[29] Woodhams R., Nishimaki H., Ogasawara G., Fujii K., Yamane T., Ishida K., Kashimi F., Matsunaga K., Takigawa M. (2013). Imipenem/cilastatin sodium (IPM/CS) as an embolic agent for transcatheter arterial embolisation: A preliminary clinical study of gastrointestinal bleeding from neoplasms. SpringerPlus.

[30] Stange R., Sahin H., Wieskötter B., Persigehl T., Ring J., Bremer C., Raschke M.J., Vieth V. (2015). In vivo monitoring of angiogenesis during tendon repair: A novel MRI-based technique in a rat patellar tendon model. Knee Surg. Sports Traumatol. Arthrosc..

